# Supplementary material for: From sequence to enzyme mechanism using multi-label machine learning
Source: BMC Bioinformatics. 2014 May 19;15:150. doi: 10.1186/1471-2105-15-150 (PMC4229970; doi:10.1186/1471-2105-15-150)
Supplement: Additional file 2 — Java code of ml2db. Additional file ml2db_code.tar.gz contains the Java source code to run the multi-label machine learning experiments and save the results to database. The code’s Javadoc is included. [file 1471-2105-15-150-S2.zip › additional file 2/ml2db/ml2db/doc/main/ml2db/mulan/mulanarff/MulanArffProperties.html]

MulanArffProperties


JavaScript is disabled on your browser.


- Overview
- Package
- Class
- Use
- Tree
- Deprecated
- Index
- Help

- Prev Class
- Next Class

- Frames
- No Frames

- All Classes

- Summary:
- Nested |
- Field |
- Constr |
- Method

- Detail:
- Field |
- Constr |
- Method


main.ml2db.mulan.mulanarff

## Class MulanArffProperties

- java.lang.Object
- - uk.ac.standrews.utils.main.Initialised
  - - main.ml2db.weka.ArffProps
    - - main.ml2db.weka.ArffPropsFromFile
      - - main.ml2db.mulan.mulanarff.MulanArffProperties

- All Implemented Interfaces:
  :   uk.ac.standrews.utils.main.Initialisable

  ---

    

  ```
  public class MulanArffProperties
  extends ArffPropsFromFile
  ```

  Class

  Version:
  :   14 Sep 2010

  Author:
  :   Luna De Ferrari ldf(at)st-andrews.ac.uk

- - ### Field Summary

    Fields

    | Modifier and Type | Field and Description |
    | --- | --- |
    | `static java.lang.String` | `XML_QUERY_PROP` The query to get the class labels for the Mulan xml file (if needed) |

    - ### Fields inherited from class main.ml2db.weka.ArffPropsFromFile

      `DATA_PROPERTIES_FILE_PATH_PROP, INSTANCE_ATTRIBUTE_QUERY_PROP, INSTANCE_CLASS_QUERY_PROP, TIMESTAMP_FIELD`
    - ### Fields inherited from class main.ml2db.weka.ArffProps

      `ARFF_DATA_PROP, ARFF_FILE_PATH_PROP, CLASS_NAME_PROP, DATASET_NAME_PROP, DESCRIPTION_PROP, NUMBER_OF_ATTRIBUTES, NUMBER_OF_CLASS_VALUES, NUMBER_OF_CORE_PROPERTIES, NUMBER_OF_INSTANCES, NUMBER_OF_STATISTICS_PROPERTIES, XML_DATA_PROP, XML_FILE_PATH_PROP`
    - ### Fields inherited from class uk.ac.standrews.utils.main.Initialised

      `m_initialisationIsCorrect`
  - ### Constructor Summary

    Constructors

    Constructor and Description

    `MulanArffProperties(java.lang.String propertiesFilePath)`
  - ### Method Summary

    Methods

    | Modifier and Type | Method and Description |
    | --- | --- |
    | `java.util.TreeSet<java.lang.String>` | `propertiesKeys()` All properties: core + statistics |

    - ### Methods inherited from class main.ml2db.weka.ArffPropsFromFile

      `coreKeys, corePropertiesAreCorrect, getNumberOfCoreProperties, getPropertiesFilePath`
    - ### Methods inherited from class main.ml2db.weka.ArffProps

      `checkPropertiesKeys, deriveXmlPath, getArffFilePath, getDbConn, getNumberOfStatsProperties, getProperties, getProperty, getXmlFilePath, propertiesAreCorrect, saveStatisticsAsProperties, statisticsKeys, statsPropertiesAreCorrect`
    - ### Methods inherited from class uk.ac.standrews.utils.main.Initialised

      `initialisationIsCorrect, setInitialisation, setInitialisationIsCorrect, setInitialisationNotCorrect`
    - ### Methods inherited from class java.lang.Object

      `equals, getClass, hashCode, notify, notifyAll, toString, wait, wait, wait`

- - ### Field Detail


    - #### XML\_QUERY\_PROP

      ```
      public static final java.lang.String XML_QUERY_PROP
      ```

      The query to get the class labels for the Mulan xml file (if needed)

      See Also:
      :   Constant Field Values
  - ### Constructor Detail


    - #### MulanArffProperties

      ```
      public MulanArffProperties(java.lang.String propertiesFilePath)
      ```
  - ### Method Detail


    - #### propertiesKeys

      ```
      public java.util.TreeSet<java.lang.String> propertiesKeys()
      ```

      **Description copied from class: `ArffProps`**

      All properties: core + statistics

      **Overrides:**
      :   `propertiesKeys` in class `ArffProps`

      Returns:


- Overview
- Package
- Class
- Use
- Tree
- Deprecated
- Index
- Help

- Prev Class
- Next Class

- Frames
- No Frames

- All Classes

- Summary:
- Nested |
- Field |
- Constr |
- Method

- Detail:
- Field |
- Constr |
- Method
